# Inheritance mode and mechanisms of resistance to imidacloprid in the house fly *Musca domestica* (Diptera:Muscidae) from China

**DOI:** 10.1371/journal.pone.0189343

**Published:** 2017-12-11

**Authors:** Zhuo Ma, Jing Li, Yi Zhang, Chao Shan, Xiwu Gao

**Affiliations:** Department of Entomology, China Agricultural University, Beijing, China; Institute of Zoology Chinese Academy of Sciences, CHINA

## Abstract

Imidacloprid is a neonicotinoid insecticide that is effective against house fly, *Musca domestica* L., which is a major pest with the ability to develop resistance to insecticides. In the present study, we investigated the inheritance mode, the cross-resistance pattern and the mechanisms of resistance to imidacloprid. A near-isogenic house fly line (N-IRS) with 78-fold resistance to imidacloprid was used to demonstrate the mode of inheritance. The overlapping confidence limits of LC_50_ values and the slopes of the log concentration-probit lines between the reciprocal F1 and F1’ progenies suggest that imidacloprid resistance is inherited autosomally in the house fly. There was incomplete dominant inheritance in the F1 and F1’ progenies, based on dominance values of 0.77 and 0.75, respectively. A monogenic inheritance model revealed that imidacloprid resistance is governed by more than one factor. Compared to the field strain (CFD), the N-IRS strain developed more cross-resistance to chlorfenapyr and no cross-resistance to chlorpyrifos and acetamiprid, but showed negative cross-resistance to beta-cypermethrin and azamethiphos. Three synergists, diethyl malate (DEM), s,s,s-tributylphosphorotrithioate (DEF), and piperonyl butoxide (PBO), showed significant synergism against to imidacloprid (4.55-, 4.46- and 3.34-fold respectively) in the N-IRS strain. However, both DEM and PBO had no synergism and DEF only exhibited slight synergism in the CSS strain. The activities of carboxylesterase (CarE), glutathione S-transferases (GSTs) and cytochrome P450 in the N-IRS strain were significantly higher than in the CSS strain. But similar synergistic potential of DEF to imidacloprid between the CSS and N-IRS strain suggested that GSTs and cytochrome P450 played much more important role than esterase for the N-IRS strain resistance to imidacloprid. These results should be helpful for developing an improved management strategy to delay the development of imidacloprid resistance in house fly.

## Introduction

The house fly, *Musca domestica* (Diptera: Muscidae) is a cosmopolitan pest of poultry and human beings [1]. It is also a transmission vector of more than 100 diseases of man and animals [2]. For decades, the management of this pest has been dependent on the application of insecticides. However, extensive and injudicious application of insecticides has resulted in the development of resistance in house fly to pyrethroid, organophosphate, carbamate and new chemical group insecticides [3–5]. Imidacloprid is a neonicotinoid insecticide that acts on the nicotinic acetylcholine receptor in the insect nervous system [6]. It was registered for house fly prevention in United States in 2004[7] and was introduced in China to control house fly in the early 1990s [8]. Imidacloprid resistance has been reported in field populations and laboratory strains of house fly [7, 9–12].

For sustainable pest management, it is necessary to understand the patterns of insecticide resistance inheritance [13, 14] and the biochemical and molecular mechanisms of resistance. Information about dominance and the number of genes involved in resistance can assist in further understanding the development of resistance [14]. Previously, the genetics of resistance to various insecticides have been explored in house fly [4, 10, 14–17]. Resistance to insecticides in insects is mostly the consequence of both target-site insensitivity in the nervous system and by increased metabolic detoxification. Cytochrome P450, carboxylesterase (CarE), and glutathione S-transferases (GSTs) are the major enzyme systems involved in the metabolic detoxification of insecticides [18]. The synergism and enzyme activities assays of each detoxification enzyme have frequently been used to examine the presence of metabolic-based resistance mechanisms [11, 16, 19]. At the molecular level, the overexpression of cytochrome P450 genes [20, 21] and the reduced expression of the nAChR subunit α2 [22] are mainly responsible for imidacloprid resistance in house fly. Currently, four nicotinic acetylcholine receptors (nAChR) subunit-encoding genes (α2, α5, α6 and β3) have been characterized from house fly; however, based on comparisons of gene sequences and expression levels there are no modifications that account for the observed differences in resistance to neonicotinoids or spinosad between the susceptible and resistant strains [23–25].

In the recent public house fly genome [26], a total of 146 cytochrome P450 genes, 33 GSTs genes and 92 esterase genes were identified. The house fly genome [26] has increased the possibilities in obtaining more contents with regard to resistance in house flies. For example, Mahmood *et al*. [27] investigate the transcriptome data of a spinosad resistant strain in relation to the house fly genome data, and they find the SNPs, CpG islands and common regulatory motifs in differentially expressed P450s, which provide a foundation to further understanding the mechanism and role of P450s in xenobiotic detoxification. Højland *et al*. [28] analyze the transcriptome data of differential expression of genes encoding metabolic detoxification enzymes, suggesting a combination of factors related to neonicotinoid and spinosad resistance.

Noteworthily, the house fly has a multifactorial sex determination system, a male determining factor (M), which is located on the X or Y chromosome or any of the five autosomes [29–32]. Sharma *et al*. [33] identified a gene, Mdmd (for *M*. *domestica* male determiner), which encodes a protein with high homology to CWC22 (nucampholin), a duplication of the spliceosomal factor gene. Targeted Mdmd disruption results in complete male-to-female transformation because of a shift from male to female expression of the downstream genes *transformer* and *doublesex*, which are conserved elements of the insect sex determination pathway.

The use of lines with same genetic background, i.e. near-isogenic lines (NILs), is very important in studies of insecticide resistance inheritance in susceptible and resistant strains to avoid interference from factors unrelated to resistance [15]. The recurrent parent and the nonrecurrent parent are used to generate NILs by crossing, backcrossing, and self-breeding. Except for the target resistant gene(s), NILs have the same genetic background as the recurrent parent [15]. In entomological studies, the use of NILs to analyze the inheritance of insecticide resistance has been reported in *Lucilia cuprina*[34] and *M*. *domestica*[15]. Based on our previous research [11], we established a near-isogenic house fly line with imidacloprid resistance to investigate the inheritance pattern of resistance, the cross-resistance to other insecticides and resistance mechanisms. Our study provides the important information on imidacloprid resistance characteristics in house flies and the information will be important for imidacloprid resistance management of house flies.

## Materials and methods

### Insects

Three strains were used in this paper: the susceptible strain (CSS), obtained from National Taiwan University in 1987, has been reared in our laboratory without exposure to any insecticides; the field strain (CFD) was collected near the Wrestling Museum at the China Agricultural University East Campus (Beijing, China) in 2007 and was maintained in the laboratory without exposure to insecticides; an imidacloprid-resistant strain (IRS) was established from the field strain (CFD) by selection with imidacloprid for 21 generations in the laboratory, and shows 80.15-fold increased resistance compared to the CSS strain. House flies were kept under standard laboratory conditions (25±1°C, 60–80% RH and a 16h:8h light:dark photoperiod) and supplied with water, sugar and milk powder.

### Chemicals

Imidacloprid (95.6%) was purchased from Dupont. Beta-cypermethrin (95%) was obtained from Suzhou Fumeishi Chemical Co., Ltd. Chlorpyrifos (98%) was obtained from Tianjin Longdeng Chemical Co., Ltd. Chlorfenapyr (98%) was obtained from Jiangsu Academy of Agricultural Sciences. Acetamiprid (90%) was provided by Jiangsu Yangnong Chemical Group Co., Ltd. Azamethiphos (95%) was supplied by Shanghai Yongyuan Chemical Co., Ltd. Piperonyl butoxide (PBO, 90%), s,s,s-tributylphosphorotrithioate (DEF, 98%) and diethyl maleate (DEM, 97%) were purchased from Chem. Service (West Chester, PA). α-Naphthyl acetate (α-NA), β-naphthyl acetate (β-NA), eserine, α-naphthol, β-naphthol, 1-chloro-2,4-dinitrobezene (CDNB), reduced glutathione (GSH), phenylmethylsulfonyl (PMSF), dithiothreitol (DTT), phenylthiourea (PTU), fast blue B salt, sodium dodecyl sulfate (SDS) and bovine serum albumin (BSA) were purchased from Sigma Chemical Co. (St. Louis, MO) at the highest purity available. The other chemicals were of analytical quality and purchased from commercial suppliers.

### Bioassays

The non-choice feeding assay with second-instar larvae of the house fly was used in the imidacloprid bioassays [11]. The breeding media, wheat bran 10 g containing imidacloprid 20 mL was used to breed twenty second-instar larvae in a disposable paper cup. A total of 60 second-instar larvae were used for each concentration. Imidacloprid was dissolved in acetone and diluted to 5–7 concentrations in water containing 0.1% TritonX-100 that gave >0% and <100% mortality. The control only contained 0.1% TritonX-100 and certain acetone. All tests were repeated three times for each concentration and performed at 25±1°C, 60–80% RH and a 16h:8h light:dark photoperiod. Mortality was assessed after 24 h. Larvae were considered dead if they made a ataxic movement when prodded with a tweezer.

### Establishment of the near-isogenic line

The method of Mu et al. [35] was used for the establishment of the near-isogenic line with a modification. For mass mating, one hundred of CSS strain female house flies and one hundred of IRS strain male house flies were put into a cage within 3 h of pupal eclosion. A self-cross was made by the offspring of the CSS strain and IRS strain. The offspring of the self-cross were referred to as F1 progeny. The second-instar larvae of the F1 progeny were tested by bioassay to determine an appropriate selection dose of imidacloprid; the remaining F1 progeny larvae were then selected with this dose until they became pupae. After pupal eclosion, a backcross was performed between the surviving F1 male house flies and female house flies of the CSS strain. The imidacloprid resistance level of the backcross progeny (BC1) was evaluated with bioassays. The BC1F1 progeny, which was made by the remaining backcross progeny (BC1) for self-cross, were selected several times with the method described above for the F1 progeny until the resistance level of the progeny BCnF1 (n is the number of backcrosses) to imidacloprid reached the resistance level of the IRS strain. The establishment of the NILs with imidacloprid resistance (N-IRS) was completed when the resistance of the self-bred progenies BCnF2 and BCnF3 had stabilized.

### Genetic crosses of CSS strain and the resistant N-IRS strain

To assess the inheritance patterns of imidacloprid resistance in house flies, it was assumed that the CSS strain and the N-IRS strain were homogeneously susceptible and resistant, respectively. Reciprocal crosses were made by mass mating between the susceptible CSS strain and the resistant N-IRS strain to obtain two lines: F1 (CSS♀×N-IRS♂) and F1’ (N-IRS♀×CSS♂). Backcrosses of the reciprocal progenies F1 and F1’ to the parental strains were conducted to obtain four lines: BC1 (F1♀×CSS♂), BC2 (F1♀×N-IRS♂), BC3 (F1’ ♀×CSS♂), and BC4 (F1’♀×N-IRS♂). The self-bred lines of the reciprocal progenies were F2 (F1♀×F1♂) and F2’ (F1’ ♀×F1’♂).

### Data analyses

Bioassay data were pooled and probit analysis was conducted with POLO software (LeOra Software Inc., Cary, NC), which can correct automatically for natural mortality, to obtain median lethal concentration (LC_50_) values and their 95% fiducial limits (FL). LC_50_ values of respective bioassays were considered significantly different on the basis of non-overlapping 95% FL. The resistance ratio was calculated by dividing the LC_50_ of N-IRS strain by the LC_50_ of the CSS strain. LC_50_ values of reciprocal cross progenies (F1 and F1’) were considered to be significantly different (P <0.05) if their 95% FL did not overlap. All biochemical assays were repeated at least three times with different preparations of enzymes. ANOVA analysis followed by the Duncans Multiple Comparison test was performed and significance is reported for P < 0.05 using the software SPSS (version 21).

The method of Stone [36] was used to calculate the degree of dominance (D) for imidacloprid resistance:

$$\text{D}=\left(2X_{F}-X_{R}-X_{S}\right)/\left(X_{R}-X_{S}\right)$$

where XF, XS, and XR are the logarithms of the LC_50_ values for the reciprocal progeny of the (F1 or F1’) susceptible and resistant strains, respectively. The case where D = 1 is indicative of complete dominance, 0 < D < 1 indicates incomplete dominance, -1 < D < 0 indicates an incomplete recessive trait, D = -1 indicates a complete recessive trait, D = 0 indicates codominance. The variance of D (σ^2^_D_) was estimated according to Preisler *et al*.[37] as follows:

$$\sigma^{2}{}_{D}=4/{\left(X_{R}-X_{S}\right)}^{2}\left\{\sigma^{2}{}_{F1}+\left[{\left(X_{F1}-X_{S}\right)}^{2}/{\left(X_{R}-X_{S}\right)}^{2}\right]\sigma^{2}{}_{R}+\left[{\left(X_{F1}-X_{R}\right)}^{2}/{\left(X_{R}-X_{S}\right)}^{2}\right]\sigma^{2}{}_{S}\right\}$$

where σ^2^_F1_, σ^2^_R_ and σ^2^_S_ are the variances of LC_50_ for the CSS, N-IRS and F1 or F1’ strains, respectively. The standard error ($SE\;=\;\sqrt{{\sigma^{2}}_{D}}$) was used to determine whether D ± 2SE was significantly different from +1 (completely dominant) to −1 (completely recessive).

The number of genes influencing imidacloprid resistance was estimated using two approaches. Following the methods of Sokal and Rohlf[38], the null hypothesis of monogenic resistance was tested on the basis of chi-square goodness-of-fit between the observed mortality and the theoretical expectation:

$${\alpha^{2}}_{i}\;=\;{\left(N_{i}-pn_{i}\right)}^{2}/pqn_{i}$$

where N_i_ is the observed number of deaths at a given dose, n_i_ is the number exposed to a given dose, p is the expected mortality estimated as described by Georghiou[39] and q = 1 − p. The null hypothesis was then tested using a chi-square table with m −1 (m is number of drug concentrations) degrees of freedom. The null hypothesis was rejected if ∑χ^2^ > χ^2^_0.05_, indicating that imidacloprid resistance was not controlled by a single gene.

In addition, the number of factors responsible for resistance was estimated by log concentration-probit line analysis of the backcross and self-bred progenies. According to Tsukamoto[40], if the log concentration-probit lines of the resistant, susceptible and their reciprocal progenies do not overlap, and where a single gene is responsible for resistance, plateaus would occur in the log concentration-probit lines of the self-bred progenies at about 25 and 75% mortality (the degree of dominance is < 0) and in the log concentration-probit lines of backcross progenies at about 50% mortality.

### Bioassay for cross-resistance and synergist study

Four-day old adult female house flies were used in bioassays by means of a non-choice feeding method for cross-resistance and synergist study[10,20]. Granulated sugar 2.0g was impregnated with 1mL of a specific concentration of insecticide or only acetone as control and the acetone in sugar was allowed to evaporate overnight before the introduction of flies into the containers. Each insecticide was dissolved in acetone at 5–7 concentrations that gave >0% and <100% mortality. Twenty of female flies were placed in a 500mL glass jam jar and fasted for 3–5 h, then the treated sugar and water (untreated) were introduced. A total of 60 female flies were prepared for each concentration. All tests were repeated three times for each concentration and performed at 25±1°C, 60–80% RH and a 16h:8h light:dark photoperiod. Mortality was assessed after 48 h and all ataxic flies were considered dead.

Synergist assays were conducted with DEF (an inhibitor of esterases), DEM (an inhibitor of GSTs) and PBO (an inhibitor of P450 monooxygenase and esterases). DEF, DEM and PBO were topically applied at the maximum sublethal dose (1 μg per fly) 1 h before the insecticide treatment [19,41]. After an hour, the treated flies were bioassayed with different concentrations of imidacloprid as stated above.

### Biochemistry assays

#### Carboxylesterase assays

CarE activities were determined with two naphthyl-substituted substrates as described by Zhang et al. [19]. Four-day-old house flies (heads were removed) were homogenized in ice-cold buffer (0.04 M phosphate buffer, pH 7.0) and centrifuged at 4°C, 10,000g for 15 min. The supernatant was filtered to remove the lipid layer, and this crude extract was used to assay enzyme activity. For each reaction, 3.6 mL substrate buffer (containing 3×10^−4^ M substrate and 3×10^−4^ eserine), 0.8 mL phosphate buffer (0.1 M, pH 7.0) and 0.2 mL crude extract were incubated at 30°C for 15 min. The reaction was stopped by the addition of 0.9 mL of fast blue B salt solution (2 parts 1% fast blue B salt and 5 parts 5% SDS). The optical density (OD) was measured at 600 nm (α-NA) or 550 nm (β-NA) to monitor hydrolysis.

#### Glutathione S-transferases assays

Activity of GSTs toward CDNB was measured as described in Habig et al[42]. Abdomens from each strain were homogenized in ice cold buffer (0.1 M phosphate buffer, pH 6.5, containing 1.0 mM EDTA and 1.0 mM DTT) and centrifuged at 4°C, 10,000g for 20 min. The filtered supernatant served as the crude enzyme source. Reaction mixtures contained GSH (final concentration 1.0 mM), enzyme homogenate buffer (0.1 M phosphate buffer, pH 6.5), and CDNB (final concentration 1.0 mM) in a total volume of 0.9 mL. The reaction was started by adding CDNB. Subsequently, the rate of change in OD at 340 nm during the initial 2 min was measured using a UV/VIS Spectrometer Lambda Bio-40 (Perkin-Elmer, USA) and converted to activity using the extinction coefficient of 9.6 mM^-1^ cm^-1^ for the reaction and the estimated protein content of the enzyme homogenate.

#### Cytochrome P450-dependent monooxygenase activity assay

Monooxygeanse activity in house flies was determined following the protocol of Lee and Scott [43]. Abdomens of four-day-old house flies from the CSS and N-IRS strains were homogenized in ice-cold homogenization buffer (0.1 M sodium phosphate buffer, pH 7.5, containing 15% glycerol, 1.0 mM EDTA, 0.1 mM DTT, 1.0 mM PTU and 1.0 mM PMSF) and centrifuged at 4°C, 10,000g for 20 min. The supernatant was filtered and this crude extract served as the source of enzyme. Following the method of Aitio[44], cytochrome P450-dependent monooxygenase activity was determined. Reaction mixtures contained Tris-HCl buffer (0.25 M, pH 8.0), NADPH (0.25 mM), BSA (0.4 mg/mL), 7-ethoxycoumarin (0.5 mM) and the crude extract in a total volume of 1.0 mL. The reaction was started by adding the crude extract. Subsequently, reaction mixtures were incubated at 30°C for 15 min. Finally, the reaction was stopped by the addition of 0.3 ml of trichloroacetic acid (5%), and the reaction mixtures were centrifuged at 4°C, 7000g for 3 min. The supernatant was transferred to a glass tube and 0.7 ml Gly-NaOH (0.6 M, pH 10.4) was added. Fluorimetric detection was done with an excitation wavelength of 380 nm and an emission wavelength of 450 nm using a Perkin Elmer LS 55 Luminescence Spectrometer (Perkin-Elmer, UK).

#### Protein assays

Protein concentration was determined by the method of Bradford [45], using bovine serum albumin as the standard.

## Results

### Establishment of the near-isogenic line

Based on LC_50_ values, the resistance of the IRS strain to imidacloprid is 80.2-fold compared with the CSS strain (Table 1). In the process of establishing the NILs the resistance ratio increased to 78.7 in the BC7F2 generation, and did not fluctuate in the following generation BC7F3, indicating that a NILs with imidacloprid resistance (N-IRS) was established.

**Table 1:** The changes of sensitivity to imidacloprid during the establishment of the N-IRS near-isogenic line.

Generation	Slope (SE)	LC_50_ (μg/g)	95% FL (μg/g)	RR^a^
**CSS**	4.96 (±1.10)	60.0	47.1–73.0	1
**IRS**	3.86 (±0.39)	4812	4294–5407	80.2
**F1**	2.12 (±0.26)	1394	1173–1722	23.2
**BC1**	2.52 (±0.26)	1390	1173–1685	23.2
**BC1F1**	2.28 (±0.38)	2617	1857–6058	43.6
**BC2**	2.79 (±0.53)	1319	1066–1914	22.0
**BC2F1**	3.11 (±0.39)	2679	2263–3149	44.6
**BC3**	1.19 (±0.22)	4437	3269–7451	73.9
**BC3F1**	1.73 (±0.26)	4076	3238–5611	67.9
**BC4**	1.34 (±0.22)	1668	724.2–2656	27.8
**BC4F1**	6.08 (±0.60)	4587	4199–4992	76.4
**BC5**	-	-	-	-
**BC5F1**	8.15 (±0.82)	4488	4194–4831	74.7
**BC6**	4.75 (±0.66)	4649	4318–5090	77.4
**BC6F1**	4.70 (±0.72)	3347	2649–3837	55.8
**BC7**	5.44 (±0.69)	3708	3432–3964	61.8
**BC7F1**	7.40 (±0.74)	4342	4124–4590	72.3
**BC7F2**	4.11 (±0.62)	4727	4358–5263	78.7
**BC7F3→N-IRS**	6.45 (±0.83)	4709	4467–4996	77.9

‘‘–” Not determined.

^a^RR, Resistance ratio calculated as LC_50_ of the BC strain/LC_50_ of the CSS strain.

### Inheritance pattern of resistance

**Fig 1:**
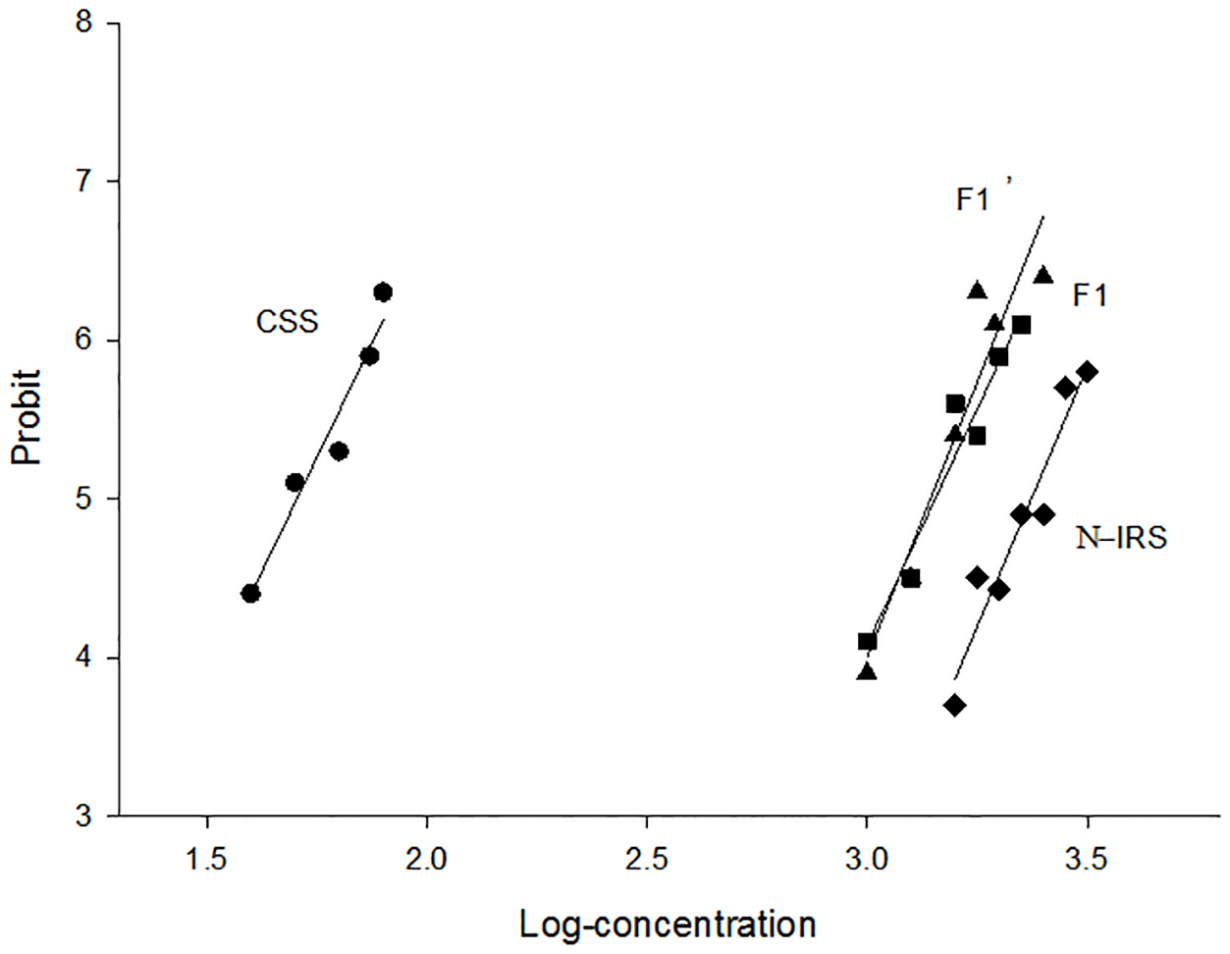
Log concentration-probit lines for (●) the susceptible CSS strain, (◆) the near-isogenic line resistant to imidacloprid (N-IRS), and their reciprocal progenies (■) F1 and (▲) F1’.

**Fig 2:**
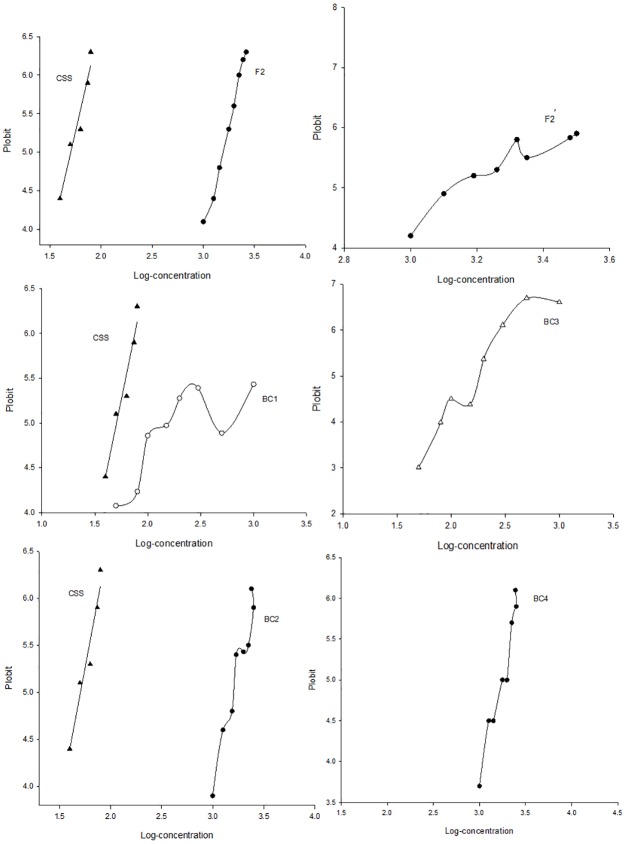
Log concentration-probit lines for the (▲) susceptible strain (CSS), backcrossed progenies (○) BC1, (◆) BC2, (Δ) BC3, (★) BC4 and self-bred progenies (●) F2, (■) F2’.

The log concentration-probit lines of parental strains and their reciprocal progenies (F1 and F1’) are straight lines (Fig 1), indicating that the CSS strain and the N-IRS strain are homogeneous for susceptibility and resistance to imidacloprid, respectively. The LC_50_ values of imidacloprid for the progeny of the reciprocal crosses (F1 and F1’) were 2822.64 and 2733.80 μg/g respectively, and there were no significant differences (overlap of 95% FL) between the LC_50_ values or the slopes of the log concentration-probit lines of the F1 and F1’ progeny (Table 2), indicating that imidacloprid resistance was inherited autosomally in house flies. There was neither sex linkage nor maternal effects in the development of resistance to imidacloprid. The values of dominance for F1 and F1’ were 0.77 (0.55–0.99) and 0.75 (0.55–0.90), respectively. The log concentration-probit lines for F1 and F1’ were intermediate between parental strains and approached the N-IRS strain. These results all demonstrate that imidacloprid resistance in house fly is an incomplete dominant trait. Based on goodness of fit tests to a monogenic model, there are significant differences between the observed and expected mortalities (∑χ^2^ = 18.28 and 27.23 for F2 and F2’, respectively, higher than the P < 0.05, df = 7 threshold value 14.07; ∑χ^2^ = 18.08 and 19.87 for BC1 and BC3, respectively, higher than the P < 0.05, df = 7 threshold value 14.07; ∑χ^2^ = 16.31 and 14.79 for BC2 and BC4, respectively, higher than the P < 0.05, df = 7 threshold value 14.07), which indicates that multiple genes are involved in development of resistance to imidacloprid (Table 3). Additionally, there were no plateaus at 50% mortality (i.e. probit 5) in the log concentration-probit lines of the backcross progenies (BC1, BC2 BC3 and BC4) nor at 25% or 75% mortality (i.e. probit 4.33 and 5.67, respectively) in the log concentration-probit line of the self-bred progenies F2 and F2’ (Fig 2), indicating that house fly resistance to imidacloprid is controlled by more than one factor.

**Table 2:** Toxicity of imidacloprid to the susceptible (CSS) and resistant (N-IRS) house fly strains and their reciprocal progenies.

Generation	Slope (SE)	LC_50_ (μg/g) (95% FL)	RR^a^	D	Var(D)
**CSS**	4.96 (±1.10)	60.0 (47.1–73.0)	1		
**N-IRS**	6.45 (±0.83)	4709 (4467–4996)	78.0		
**F1 (CSS♀×N-IRS♂)**	6.03 (±0.75)	2823 (2579–3042)	47.0	0.77	0.22
**F1’ (N-IRS♀×CSS♂)**	7.54 (±0.81)	2733 (2519–2928)	45.2	0.75	0.20

^a^RR, Resistance ratio calculated as LC_50_ of the F1 (F1’) strain/LC_50_ of the CSS strain

**Table 3:** Toxicity of imidacloprid and chi-square analysis of monogenic inheritance of imidacloprid resistance in house flies.

Generation	Slope (SE)	LC_50_ (μg /g) (95% FL)	RR^a^	χ^2^(df)^b^
**F2 (F1♀×F1♂)**	5.59(±0.57)	3088(2878–3284)	51.4	18.3(7)
**F2’(F1’ ♀×F1’♂)**	2.70(±0.44)	3003(2212–3553)	50.0	27.2(7)
**BC1 (F1♀×CSS♂)**	1.10(±0.15)	427(266–666)	7.11	18.1(7)
**BC2 (F1♀×N-IRS♂)**	3.97(±0.48)	3357(3051–3646)	55.9	16.3(7)
**BC3 (F1’ ♀×CSS♂)**	2.99(±0.27)	345(283–422)	5.76	19.8(7)
**BC4 (F1’ ♀×N-IRS♂)**	4.51(±0.48)	3564(3215–3907)	59.4	14.8(7)

^a^RR, Resistance ratio calculated as LC_50_ of the F2 (F2’) or BC strain/LC_50_ of the CSS strain.

^b^ df, degrees of freedom.

### Patterns of cross-resistance

The N-IRS strain showed significant cross-resistance to chlorfenapyr (RR = 8.56 fold; non-overlapping 95% FL) and no cross-resistance to acetamiprid and chlorpyrifos (overlapping 95% FL), but showed negative cross-resistance to beta-cypermethrin (LC_50_ decreased from 211 to 21.1μg/g) and azamethiphos (LC_50_ decreased from 50 to 14.1μg/g). (Table 4).

**Table 4:** Cross-resistance of the N-IRS strain to other insecticides.

Insecticide	Strain	Slope (SE)	LC_50_ (μg /g) (95% FL)	RR^a^	RR^b^
**Imidacloprid**	CSS	3.76 (±0.59)	162 (142–185)	1	
	CFD	2.68 (±2.68)	1535 (1220–1835)	9.48	1
	N-IRS	1.33 (±0.41)	32690 (24776–68759)	201	21.3
**Acetamiprid**	CSS	1.63 (±0.30)	102 (78.0–150)	1	
	CFD	1.51 (±0.20)	1710 (1345–2295)	16.7	1
	N-IRS	1.46 (±0.35)	2081 (993–2826)	20.3	1.21
**Chlorfenapyr**	CSS	1.74 (±0.26)	22.1 (15.6–41.1)	1	
	CFD	3.56 (±0.43)	6.20 (5.05–7.40)	0.28	1
	N-IRS	3.67 (±0.44)	53.1 (43.4–63.3)	2.40	8.56
**Beta-cypermethrin**	CSS	2.39 (±0.29)	1.16 (0.95–1.44)	1	
	CFD	1.76 (±0.26)	211 (163–289)	181.9	1
	N-IRS	1.56 (±0.20)	21.1 (13.1–38.2)	18.26	0.10
**Chlorpyrifos**	CSS	2.46 (±0.34)	23.4 (17.2–28.7)	1	
	CFD	2.52 (±0.33)	67.5 (51.5–101)	2.89	1
	N-IRS	3.04 (±0.53)	42.3 (34.1–68.4)	1.81	0.63
**Azamethiphos**	CSS	2.97 (±0.47)	11.5 (9.81–14.4)	1	
	CFD	1.85 (±0.29)	50 (33.7–100)	4.35	1
	N-IRS	3.27 (±0.46)	14.1 (10.2–17.1)	1.22	0.28

^a^RR, Resistance ratio calculated as LC_50_ of the N-IRS strain/LC_50_ of the CSS stain.

^b^RR, Resistance ratio calculated as LC_50_ of the N-IRS strain/LC_50_ of the CFD stain.

The datas of CFD are cited from Li *et al*[13].

### Synergism of PBO, DEM and DEF

Bioassay results showed that two synergists, PBO and DEM did not have significant synergistic effects on imidacloprid toxicity in the CSS strain, however, they have significant synergistic effects on imidacloprid toxicity in the N-IRS strain. The synergistic ratios were 4.55 for DEM and 3.34 for PBO. Similar synergistic potential of DEF to imidacloprid between the two strains was observed. The synergistic ratio of DEF to imidacloprid was 2.43 and 4.46 for the CSS and N-IRS strain, respectively. (Table 5).

**Table 5:** Synergistic effects of DEF, DEM and PBO on imidacloprid toxicity in the N-IRS and CSS strains.

Strain	Compound	Slope (SE)	LC_50_ (μg /g) (95% FL)	SR^a^
**CSS**	Imidacloprid	3.76 (±0.59)	161 (142–185)	1
	+DEF	1.05 (±0.32)	66.7 (8.10–138)	2.43
	+DEM	1.81 (±0.39)	124 (96.4–164)	1.30
	+PBO	2.30 (±0.43)	127 (104–167)	1.27
**N-IRS**	Imidacloprid	1.33 (±0.41)	32690 (24776–68759)	1
	+DEF	0.86 (±0.21)	7336 (3962–11081)	4.46
	+DEM	1.07 (±0.21)	7184 (3245.50–11649)	4.55
	+PBO	1.44 (±0.22)	9785 (6836–13635)	3.34

^a^SR, Synergism ratio calculated as LC_50_ of imidacloprid/LC_50_ of PBO or DEF or DEM + imidacloprid.

### Biochemical assays

The specific activities of CarE, GSTs and P450 were significantly higher (P<0.05) in the N-IRS than in the CSS strain (Table 6). The activity of P450 in the N-IRS strain was 4.58 times higher than in the CSS strain, the activity of GSTs in the N-IRS strain was 2.40 times higher than in the CSS strain. CarE hydrolysis of α-NA and β-NA, in the N-IRS was only 1.28 and 1.45 times higher than in the CSS strain, respectively, although this difference was significant.

**Table 6:** Comparison of carboxylesterase (CarE), glutathione S-transferases (GSTs) and cytochrome P450 (P450) activities between the CSS and N-IRS strains.

Strains	CarE activity^1^		GSTs activity^2^	P450 activity^3^
	α-NA	β-NA	CDNB	7-ECOD
**CSS**	90.5±2.80a	39.3±0.64a	55.4±3.86a	6.74±0.32a
**N-IRS**	116±6.54b	56.8±5.57b	133±7.44b	30.9±3.83b

Different letters in each column indicate statistical differences based on ANOVA analysis followed by Duncan’s Multiple Comparison test (P < 0.05).

^1^ CarE activity was calculated using nmolmg protein^-1^min^-1^

^2^ GSTs activity was calculated using nmolmg protein^-1^min^-1^

^3^ P450 activity was calculated using pmolmg protein^-1^min^-1^

## Discussion

In this paper, a near-isogenic house fly line (N-IRS) resistant to imidacloprid was used to determine that imidacloprid resistance is inherited as a polygenic, autosomal, and incompletely dominant trait. Previously, Kavi *et al*.[10] cultured an imidacloprid-resistant strain of house fly with 2300-fold and 130-fold increased resistance in females and males, respectively. In this strain, the inheritance of imidacloprid resistance was an autosomal trait, with incomplete dominance in males and intermediate dominance in females. Khan *et al*.[12] reported that an imidacloprid-selected population of house fly (Imida-SEL) had 106-fold higher resistance, and imidacloprid resistance was inherited as an autosomal, polygenic and incompletely recessive trait. In this population, the values of dominance for the F1 and F1’ progeny were 0.53 and 0.31, respectively. However, in our study, dominance values for the F1 and F1’ progeny were 0.77 and 0.75, respectively, indicating that imidacloprid resistance is incompletely dominant. Unlike the N-IRS strain used in our study, this Imidacloprid-SEL population was derived from a field-collected strain that was under laboratory successive selection with imidacloprid for 13 generations. This indicates that different selection histories and genetic backgrounds may be responsible for these differences in dominance. Khan *et al*[12] found that imidacloprid resistance was incompletely recessive (D_ML_ = 0.08) at the highest tested dose (256 μg /ml) and incompletely dominant (D_ML_ = 0.75) at the lowest tested dose (16 μg /ml). Therefore, although it is generally believed that the dominance level for a particular character is a fixed parameter, it may be affected by environmental conditions and genetic background [46].

It is important to understand how resistance is inherited for determining how to effectively use an insecticide to control a particular pest [47]. The degree of dominance may play a significant role in the expression and distribution of the resistance genes. If dominant genes control insecticide resistance, it will make chemical control more difficult due to the fact that heterozygotes are also resistant [48]. Resistance controlled by recessive genes may develop more slowly than resistance controlled by dominant genes because resistant phenotypes (RR and RS) were more than susceptible phenotypes (only SS) [48, 49]. The number of genes involved in resistance and their dominance characteristics have a direct bearing on the speed of evolution of resistance [17]. According to computer modeling, resistance controlled by two or more genes would develop more slowly than resistance controlled by a single gene [50]. Therefore, our research results reveal the potential for rapidly increased imidacloprid resistance in the future, and the reasonable use of imidacloprid will be needed for retaining its efficacy for as long as possible.

In the present study, we found that the N-IRS strain had higher cross-resistance to chlorfenapyr (8.56-fold) which targeting on different sites to imidacloprid. Elevated metabolic detoxification might account for this cross-resistance. If an insecticide selects specific isozymes that can act on different insecticides, cross-resistance with other insecticides is likely [51]. Lacking of cross-resistance to neonicotinoid (acetamiprid) and organophosphate (chlorpyrifos) might provide an opportunity to use these compounds in rotation or as alternatives for controlling the house fly. No cross-resistance between imidacloprid and chlorpyrifos was also observed by Abbas *et al*. [52]. Interestingly, there were negative cross-resistance to beta-cypermethrin and azamethiphos. This phenomenon could be the result of a gradual disappearance of beta-cypermethrin and azamethiphos resistance due to the fitness cost might be high for maintaining the resistant gene(s). Previously, in a similar study by Li *et al*.[11], the field strain after selection with imidacloprid for 21 generations showed 140-fold resistance to imidacloprid (IRS) when compared with the susceptible strain (CSS). The IRS strain developed relative order of the cross-resistance to these five insecticides (chlorfenapyr > beta-cypermethrin > azamethiphos > chlorpyrifos > acetamiprid) with the field strain as control. One possible explanation for this observation is that the different genetic background between the IRS and the N-IRS strain.

There was significant synergism between PBO and DEM on imidacloprid in the N-IRS strain but not in the CSS strain, consistently, the activities of GSTs and P450 in the N-IRS strain were significantly higher than in the CSS strain. The activity difference of CarE between the N-IRS and the CSS strains was significant, however, the effect of DEF on imidacloprid toxicity was implicated by synergism assay in either the N-IRS or CSS strains and the synergistic potential of DEF to imidacloprid between the two strains was similar. The above results of both the synergism and biochemical assays indicated that imidacloprid resistance was likely associated with esterase, GSTs and cytochrome P450, but GSTs and cytochrome P450 played more important role than esterase in the N-IRS strain resistance to imidacloprid. Previously, it was shown that cytochrome P450 monooxygenase is involved in the imidacloprid resistance of house fly [11,20] and cytochrome P450-meditated resistance due to P450 gene(s) overexpression is a major mechanism for high level resistance to neonicotinoid[20,21]. Markussen and Kristensen [20] reported that the over-expression of cytochrome P450 genes (CYP6A1, CYP6D1, CYP6D3) contribute to imidacloprid resistance in two field populations of the house fly, 766b and 791a, developed 20–140 times resistance to imidacloprid. CYP6G4 has been proven to be a major insecticide resistance gene related to neonicotinoid resistance, by overexpression of CYP6G4 in the resistant strain in comparison with the susceptible reference strain WHO-SRS [21].

In conclusion, the imidacloprid resistance is inherited as a polygenic, autosomal, and incompletely dominant trait. The N-IRS strain demonstrated significant cross-resistance to chlorfenapyr and no cross-resistance to acetamiprid and chlorpyrifos, but showed negative cross-resistance to beta-cypermethrin and azamethiphos. Moreover, enhanced activities of CarE, GSTs and cytochrome P450 enzymes are likely to be associated with imidacloprid resistance. Despite the growing number of documented cases of resistance, there were no counts of the house fly resistance to imidacloprid in China, the above results could be beneficial in the development of a proactive resistance management plan to preventing imidacloprid resistance crisis become severe in the future. In addition, measures will be done to examine the inheritance pattern and resistance frequencies in future regular monitoring to develop a scientific and comprehensive strategy against house flies. Nevertheless, our conclusions were necessarily tenuous and further studies were required the information about molecule biology properties of GSTs- and P450-mediated imidacloprid resistance in house flies.
